# Phenolic Compounds Characteristic of the Mediterranean Diet in Mitigating Microglia-Mediated Neuroinflammation

**DOI:** 10.3389/fncel.2018.00373

**Published:** 2018-10-23

**Authors:** Ruth Hornedo-Ortega, Ana B. Cerezo, Rocío M. de Pablos, Stéphanie Krisa, Tristan Richard, M. Carmen García-Parrilla, Ana M. Troncoso

**Affiliations:** ^1^MIB, Unité de Recherche Oenologie, EA4577, USC 1366 INRA, ISVV, Unive. de Bordeaux, Bordeaux, France; ^2^Departamento de Nutrición y Bromatología, Toxicología y Medicina Legal, Área de Nutrición y Bromatología, Facultad de Farmacia, Universidad de Sevilla, Seville, Spain; ^3^Departamento de Bioquímica y Biología Molecular, Facultad de Farmacia, Universidad de Sevilla, Seville, Spain

**Keywords:** neuroinflammation, microglia, phenolic compounds, wine, olive oil, Mediterranean diet

## Abstract

Neuroinflammation is a pathological feature of quite a number of Central Nervous System diseases such as Alzheimer and Parkinson’s disease among others. The hallmark of brain neuroinflammation is the activation of microglia, which are the immune resident cells in the brain and represents the first line of defense when injury or disease occur. Microglial activated cells can adopt different phenotypes to carry out its diverse functions. Thus, the shift into pro-inflammatory/neurotoxic or anti-inflammatory/neuroprotective phenotypes, depending of the brain environment, has totally changed the understanding of microglia in neurodegenerative disease. For this reason, novel therapeutic strategies which aim to modify the microglia polarization are being developed. Additionally, the understanding of how nutrition may influence the prevention and/or treatment of neurodegenerative diseases has grown greatly in recent years. The protective role of Mediterranean diet (MD) in preventing neurodegenerative diseases has been reported in a number of studies. The Mediterranean dietary pattern includes as distinctive features the moderate intake of red wine and extra virgin olive oil, both of them rich in polyphenolic compounds, such as resveratrol, oleuropein and hydroxytyrosol and their derivatives, which have demonstrated anti-inflammatory effects on microglia on *in vitro* studies. This review summarizes our understanding of the role of dietary phenolic compounds characteristic of the MD in mitigating microglia-mediated neuroinflammation, including explanation regarding their bioavailability, metabolism and blood–brain barrier.

## Introduction

Among age-related diseases, neurodegenerative disorders are the most prevalent. According to the World Health Organization (WHO), worldwide, around 50 million people have dementia, and every year, there are nearly 10 million new cases. Also the proportion of the general population aged 60 and over with dementia at a given time is between 5 to 8 per 100 people and the total number of people with dementia is expected to increase to 82 million in 2030 and 152 in 2050 (WHO, 2015, 2017). Among them, Alzheimer’s disease (AD) and Parkinson’s disease (PD), are most common and of great concern since they are chronic and progressive and affect a significant portion of the aged population. AD is the most common form of dementia, accounting for around 60–70% of total cases (WHO, 2017). At the same time their poor diagnosis and lack of effective treatment worsens the problem (Figueira et al., 2017; Pennisi et al., 2017). Since their prevalence remains growing, they currently represent a challenge for society and healthcare systems (Deak et al., 2015; Peña-Altamira et al., 2017). Although age is the main risk factor for dementia, there are other recognized risk factors, quite a number of them related with diet such as mid-life hypertension, obesity or unbalanced diets (WHO, 2018). Hence, neurodegenerative disorders are recognized to be complex, progressive and multifactorial. At the same time genetic factors are associated since familial and sporadic forms are described with lifestyle and environmental factors involved (Nicolia et al., 2014; Peña-Altamira et al., 2017).

Pharmacological treatments for AD and PD currently available have the potential to delay the progression or even reduce the symptoms at a certain level (Pennisi et al., 2017). At the same time, they have common pathological features such as oxidative stress, abnormal protein aggregation, inflammation and apoptosis of neurons (Angeloni et al., 2017). In this context and due to the limited efficacy of pharmacological treatment and the multifactorial nature of these disorders, a multifaceted approach seems appropriate. Diet interventions are a promising approach to prevent and delay the progression, with so far an important body of evidence and experimental support (Almeida et al., 2016; Figueira et al., 2016; Angeloni et al., 2017; Pennisi et al., 2017; Pistollato et al., 2018).

Although prevalence data of neurodegenerative diseases within the EU-28 countries do not support lower prevalence figures for Mediterranean countries (Alzheimer Europe, 2013), MD is still widely recognized for its healthy pattern (Castro-Quezada et al., 2014). According to the last data provided by Eurostat (Eurostat, 2016), life expectancy at age 65 in Mediterranean countries is significantly higher than the average for EU-28 countries. Since neurodegenerative diseases are strongly aged-related, it is not surprising that its overall prevalence values in Mediterranean countries remain indistinct than another EU-28 countries.

Mediterranean diet has been proposed as a healthy dietary pattern with increasing evidence supporting its beneficial effects toward quite a number of age-related pathologies, among them neurodegenerative disorders and cognitive dysfunctions (Féart et al., 2009, 2010, 2013; Tangney et al., 2011). A number of studies have shown how adherence to the MD pattern is associated with a reduction on cognitive decline and a reduced risk of dementia, AD and PD (Scarmeas et al., 2006, 2009; Di Giovanni, 2009; Alcalay et al., 2012; Gardener et al., 2012; Singh et al., 2014; Casamenti et al., 2015; Safouris et al., 2015; Anastasiou et al., 2017). In addition, MD dietary patterns (a vegetable-based diet and a moderate alcohol intake, especially wine) have been also observed in the so-called “Blue Zones.” These zones are population areas [Sardinia (Italy), Okinawa (Japan), Loma Linda (California), Nicoya Peninsula (Costa Rica) and Icarian (Greece)] which share apart of similar dietary patterns to MD, other special particularities as a stress free and active life-style (regular physical activity) and a familial, social and spirituality life (Buettner and Skemp, 2016). “Blue Zones” have been object of investigation due to the high and exceptional longevity (centenarians/non-agenarians) of their population (Poulain et al., 2013). In fact, it has been observed that older Blue Zone Sardinians present fewer cognitive failures in comparison with the population of other Italian zone (Lombardy). This observation has been related with the presence of a superior working memory performance and lower levels of depressive symptoms associated to the life style pattern including the diet (Fastame et al., 2014a,b, 2015; Fastame and Penna, 2014).

This potential of MD in preventing neurodegenerative disorders has been mainly related with its high content in plant foods: fruits and vegetables and olive oil, sources of an array of bioactive compounds (Morris et al., 2006; Scarmeas et al., 2006, 2009; Féart et al., 2010, 2013; Kelsey et al., 2010; Nooyens et al., 2011; Scapagnini et al., 2011; Tangney et al., 2011; Alcalay et al., 2012; Davinelli et al., 2014; Angeloni et al., 2017; Figueira et al., 2017). Bioactive compounds comprise an heterogeneous group of thousands of molecules present mainly in plant foods and also known as phytochemicals. They can be classified into a number of groups, depending on authors, being carotenoids, phytosterols, polyphenolic compounds and sulfur compounds the most abundant.

Research on the protective effect of food polyphenols toward the prevention of neurodegenerative disorders has been extensive in recent years (Singh et al., 2008; Joseph et al., 2009; Dixon and Pasinetti, 2010; Spencer, 2010; Jones et al., 2012; Vauzour et al., 2018). Virgin olive oil (VOO) and wine are two characteristic polyphenol-rich food items of the MD. Among polyphenols, stilbenes seem to be one of the most promising groups of compounds due to its bioactivity, with red wine being the main source of the most abundant dietary stilben: resveratrol (RV). Most important polyphenols in VOO are tyrosol derivates, showing hydroxytyrosol (HT) and oleuropein (OLE) the most relevant neuroprotective effects (Daccache et al., 2011; Barbaro et al., 2014; Casamenti et al., 2015; Rigacci and Stefani, 2015). Additionally, HT has also been found in both red and white wine in significant amounts (Fernández-Mar et al., 2012). In fact, dietary supplementation with extra virgin olive oil (EVOO) improves behavioral deficits in aging rats (Pitozzi et al., 2010). Moreover the longitudinal “Three city study” found an association between protective effects of the MD and cognition in an elderly population (Berr et al., 2009). Several clinical trials and population studies show olive phenolic compounds as the main responsible for the protective effects against aging-associated cognitive disorders and neurodegenerative diseases such AD, with a simultaneous improvement of cognitive performance (Scarmeas et al., 2006; Valls-Pedret et al., 2012, 2015; Rigacci and Stefani, 2015; Rodríguez-Morató et al., 2015; Casamenti and Stefani, 2017; Loughrey et al., 2017). Despite the evidence supporting the potential benefit of MD, the epidemiological evidences are scarce and need further critical discussion.

Polyphenols are an extensive group of molecules, whose number is of several thousands, and encompass very different structures, concentrations in food and beverages and bioactivities. Usually, after ingestion only a minor part is readily absorbed in the upper intestine, most frequently they are hydrolyzed and metabolized prior to their absorption (Bolca et al., 2013; Faria et al., 2014). The non-absorbed portion reaches the colon where it is extensively utilized by gut microbiota yielding low molecular weight compounds, mainly low molecular weight fatty acids (Manach et al., 2004; Crozier et al., 2009; Selma et al., 2009; Laparra and Sanz, 2010; Williamson and Clifford, 2010; Rodriguez-Mateos et al., 2014).

One main issue is if these compounds are able to pass the blood brain barrier (BBB), reaching significant concentrations in brain. Different families of dietary polyphenols present neuroprotective properties but they need a good permeability across the BBB to be really effective (Bisht et al., 2010; Figueira et al., 2017). At the same time low absorption rates and rapid metabolism and excretion could limit their efficacy (Almeida et al., 2016). Nevertheless, the question of the actual dose reaching the target tissues remains uncertain (Vauzour, 2012).

The mechanisms by which polyphenols are able to prevent and counteract neurodegenerative diseases include interfering with amyloid aggregation, reducing oxidative stress and regulating signaling pathways and cytokines expression, with a marked effect on reducing inflammation (Ramassamy, 2006; Essa et al., 2012; Martínez-Huélamo et al., 2017; Sarubbo et al., 2017). In fact, inflammatory markers, many of them derived from activated microglia, are widely present in neurodegenerative diseases and polyphenols have been proposed as active agents having anti-inflammatory effects on microglia (Sundaram and Gowtham, 2012; Peña-Altamira et al., 2017; Cayero-Otero et al., 2018). Despite the broad evidence supported by both *in vitro* and *in vivo* studies, it is worth highlighting that we should be cautious when extrapolating the findings based on cell culture or animal research to the human disease. Nevertheless, these approaches are fundamental to underpin the effects observed in human intervention studies.

Recently, Weber (2015) has analyzed the inherent benefits and drawbacks of both *in vitro* and *in vivo* methodologies used for assessing neuroprotection. In summary, *in vitro* approaches make possible to conduct a rapid screen (and test different concentrations) to assess the potential effects of bioactives and represent a good model to glimpse the cellular effects and discern the mechanism of action. Moreover, *in vitro* techniques can be used to study protective activities over the course of a few weeks, compared to *in vivo*, that may need several months. However, the compounds are sometimes tested at concentrations that are not achieved in nervous system tissue and without taking account the different human physiological processes such digestion, metabolism and the role of gut microbiota. In addition, many cellular lines are genetically modified and consequently it may not represent the real characteristics of cells in the brain. Concerning *in vivo* methodology, it allows deepening to determine more adequately the protective effects of bioactives or even their metabolites in the different brain areas and enables to determine the extent dietary compounds that can pass to the brain (Weber, 2015). However, one of the most current important challenge for neurodegenerative research is to develop better animals models that properly reflect both disease etiology and progression (Franco and Cedazo-Minguez, 2014), that can replace the based massive overexpression protein animal models that are not fitted for this goal.

In despite of all above mentioned, unfortunately, it has been described that only a third of the preclinical animal research are later translated at the level of human randomized trials (Hackam and Redelmeier, 2006).

This review summarizes the evidence of the role of certain dietary phenolic compounds characteristic of the MD (stilbenes, HT and OLE) in mitigating microglia-mediated neuroinflammation by inhibiting key signaling pathways.

## Shift in Microglial Phenotypes as a Target to Combat Neurodegenerative Disorders

As stated above, neuroinflammation is a common feature shared by most neurodegenerative disease, such as AD and PD.

Alzheimer’s disease, the most common form of dementia in the elderly, is characterized by the accumulation of amyloid-β (Aβ) both in the brain parenchyma (amyloid plaques) and blood vessels (cerebral amyloid angiopathy), and by the presence of neurofibrillary tangles (Wuwongse et al., 2010). AD is characterized by a progressive cognitive decline, memory loss and atrophic changes in some brain areas in response to massive neuronal death and synaptic degeneration (Wenk, 2003; Wuwongse et al., 2010). There is strong evidence demonstrating a close correlation between Aβ accumulation and neuroinflammation, and the active role of the immune system in the etiology of AD. Aβ is toxic to neurons by itself, which in turn overactivates microglia (Yankner et al., 1990; Heneka et al., 2010) with the subsequent deleterious effect to neurons (Block et al., 2007).

PD is the second most prevalent neurodegenerative disease, affecting approximately 1–3% of the population (Obeso et al., 2000). This neurodegenerative disorder is characterized by a slow and progressive degeneration of dopaminergic neurons in the SN (Obeso et al., 2000). This loss of dopamine is responsible for many of the symptoms that accompany the disease, including motor dysfunction, mood alterations and cognitive impairment (Olanow et al., 2003). Evidence of neuroinflammation as an underlying process in PD has been accumulating since the presence of activated microglia in the substantia nigra (SN) of PD patients was first reported by McGeer et al. (1988). This increase of activated microglia is accompanied by an increase in the expression of pro-inflammatory cytokines (Tansey et al., 2007; Hirsch and Hunot, 2009).

Neuroinflammation is mainly carried out by microglia cells, the macrophages of the central nervous system (CNS). Although very similar in terms of morphology and functions, peripheral macrophages and microglia have distinctive characteristics among which are their origin, functions and markers. Besides, macrophages/microglia have diverse functions that range from fighting bacterial infection to tissue regeneration and wound healing. The diverse functions of microglial cells in the CNS are mirrored by equally diverse phenotypes. A classical model of pro-inflammatory/M1 *versus* an anti-inflammatory/M2 microglia has been extensively used. However, the complex and different functions of microglial cells can only be explained by the existence of varied and plastic microglial phenotypes mediated by distinct gene expression programs and a network of molecular pathways that relay environmental signals via signaling cascades (Amici et al., 2017). Therefore, M1 and M2 are just the extremes of a broad spectrum of phenotypes that cover the different functions of microglia. These different phenotypes can be achieved by stimulating microglial cells with different compounds. Hence, when stimulated with lipopolysaccharide (LPS) (a bacterial cell wall product of Gram-negative bacteria) and interferon gamma (IFN-γ), macrophages/microglia has long been known as classically activated or M1 (Martinez and Gordon, 2014), while when activated with IL-4 macrophages/microglia show an alternative activated phenotype or M2. In order to standardize the nomenclature and facilitate the communication of macrophage/microglia data, a novel nomenclature has been proposed in which the letter M is followed by a parenthesis that includes the stimuli used for activation (Murray et al., 2014). The knowledge of the molecular programs that control the inflammatory phenotypes *versus* resolution provides a unique opportunity to find new targets that allow modulating these phenotypes and, therefore, controlling the excessively inflammatory responses that accompany neurodegenerative diseases. The knowledge of these molecular mechanisms is greatly advancing in recent years.

Functionally, M1 microglia is responsible for fighting infections, for which it adopts a clear pro-inflammatory phenotype with microbicidal, antigen-presenting and immune-enhancing functions. This type of microglia is characterized by the production of NO by the iNOS, encoded by the *Nos2* gene; (MacMicking et al., 1997; Arnold et al., 2014) and by the expression of inflammatory chemokines and cytokines, such as interleukin IL-6, IL-12, IL-1β, IL-23, and TNF-α that attract new cells of the immune system to the site of infection (Mosser and Edwards, 2008; Murray et al., 2014). In the context of neurodegenerative disease this phenotype produces harmful effects in the neuronal population.

When neutrophils undergo apoptosis and microglia switch to a resolution/M2 phenotype, the initial acute inflammation evolves to a resolution phase (Serhan et al., 2014). This resolution/wound healing phase is mediated by lipid mediators, such as classical eicosanoids, phospholipids and sphingolipids, endocannabinoids and specialized proresolving mediators (Chiurchiù and Maccarrone, 2016), that promote the switch of microglia to the M2 phenotype (Bosurgi et al., 2017). Resolution/M2 microglia suppresses IL-12 secretion and induces the release of IL-10, TGB-β, IL-1R antagonist and decoy IL-R II (Brancato and Albina, 2011). Besides, these microglial cells induce the expression of arginase-1 instead of iNOS, switching arginine metabolism from production of NO to ornithine, and also increase polyamines production for extracellular matrix and collagen synthesis (Gordon and Martinez, 2010). This phenotype promotes the neuroregeneration and tissue repair.

Taking into account the importance of inflammation in neurodegenerative diseases, the scientific community is searching for new strategies that may induce a shift in microglial cells from inflammatory and neurotoxic phenotype to an anti-inflammatory and neuroprotective one. In this sense, several compounds have shown immunomodulatory properties, making them possible candidates for co-adjuvant therapies to treat neurodegenerative diseases.

## RV and Other Stilbenes: Bioavailability, Pharmacokinetics and Blood–Brain Barrier Permeability

**FIGURE 1:**
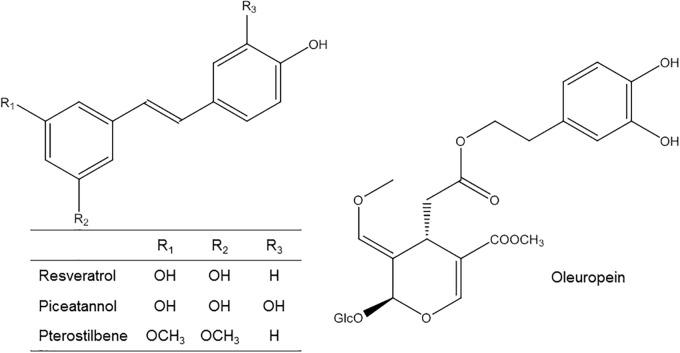
Chemical structure of RV and derivatives and OLE.

Stilbenes such as RV (Figure 1) are found in many plant species including *Arachis hypogaea* (peanut), *Vitis vinifera* (grape wine) and many tree species such as *Picea*, *Pinus*, and *Eucalyptus* (REF BD stilbenes). Stilbenes are synthetized in plants by the condensation reaction of 4-coumaroyl CoA and 3 molecules of malonyl CoA under the action of stilbene synthase (STS). STS is the key specific enzyme of stilbene-producing plants (Soleas et al., 1997). The distribution of STS and stilbenes is organ-specific and tissue-specific (Wang et al., 2010). Stilbene production is increased in response to abiotic and biotic stresses such as UV-radiations, hydric stress or infectious diseases. Concerning dietary sources, stilbenes have been identified in peanuts, blueberries, and cranberries (Neveu et al., 2010). Nevertheless grape skins and red wine constitute the main primary dietary sources in the human diet (Weiskirchen and Weiskirchen, 2016). The levels of RV, the most studied stilbene, range from non-detectable levels to 29.2 mg/L in red wines (Stervbo et al., 2007). In addition to RV, red wine contains several other stilbenes such piceid (its glucoside), piceatannol and its glucoside astringin, pterostilbene, or viniferins (Pezet et al., 1994; Vitrac et al., 2005; Guebailia et al., 2006). These compounds are present as constitutive compounds of the woody organs and as induced substances in leaves and grape berries acting as phytoalexins (Vrhovsek et al., 2012; Gabaston et al., 2017).

The possible beneficial effect on human health of stilbenes depends heavily on their absorption, bioavailability, and metabolism. First at all, due to its structure, RV is poorly soluble in water (<0.05 mg/mL) which could affect its bioavailability. Organic solvents such as alcohol increase its solubility. At least 70% of resveratrol ingested is absorbed, and readily metabolized to form mainly glucuronide and sulfate derivatives (Fernández-Mar et al., 2012). A rapid passive diffusion of RV and the formation of complexes with membrane transporters have been reported at the intestinal level (Delmas et al., 2011). Phase II metabolism of both resveratrol and its metabolites takes place at hepatic level (Kaldas et al., 2003; Li et al., 2003). Furthermore, it is known that RV can induce its own metabolism by increasing the activity of phase II detoxifying enzymes (Lançon et al., 2004). In addition, it has been described that RV can undergoes some returning cycles to the small intestine due to the enterohepatic transport (Crozier et al., 2009). Therefore, three different forms: glucuronide, sulfate or free RV are the main forms found in the bloodstream. At the same time, only trace amounts of unchanged RV have been detected in plasma (Walle et al., 2004). The main metabolites excreted in urine and feces (probably by enterohepatic cycle) have been RV sulfate and RV glucuronide derivatives (Marier et al., 2002). Besides, RV and its metabolites have been found distributed among various organs, such as liver, kidney, lung, brain, small intestinal mucosa, and colonic mucosa (Vitrac et al., 2003; Wenzel and Somoza, 2005). Additionally, mention should also be made of the significance of formation of RV metabolites by gut microbiota. Dihydroresveratrol (Walle et al., 2004), 3,4′-dihydroxy-*trans*-stilbene and 3,4-dihydroxybibenzyl (lunularin) (apart from glucuronides and sulfates) have been identified as RV metabolites after microbiota biotransformation with human fecal material (Bode et al., 2013). Bioavailability of unchanged RV is very low (almost zero) due to the rapid and extensive biotransformation, despite that it shows several *in vivo* activities (mouse and humans) (Walle et al., 2004; Gambini et al., 2015; de Vries et al., 2018).

Concerning other stilbenes, piceatannol (Figure 1) has been identified in wine and tea (Neveu et al., 2010). Its absorption, bioavailability and metabolism seem to be similar to that of RV (Piotrowska et al., 2012). *In vivo* experiments indicated that piceatannol is a metabolite of RV (Niles et al., 2006) an that it seems to be absorbed from the intestine after oral ingestion and rapidly metabolized to both glucuronidation and sulfation. Recent studies have identified also piceatannol metabolites such as *O*-methyl conjugates and isorhapontigenin (methylated derivative of piceatannol) and its conjugates demonstrating that piceatannol is not only a RV metabolite (Setoguchi et al., 2014). In addition the same authors found that piceatannol was absorbed twofold greater in the intact form than RV. All these data suggests that piceatannol has a more complicated metabolic pathway due in part to the presence of a catechol ring, which enables methylation and increases the number of pathways available during its metabolism (Setoguchi et al., 2014). Nevertheless, more studies are necessary to gain more knowledge on piceatannol metabolism and to investigate its biological properties.

Pterostilbene (Figure 1) is a dimethyl ether analog of RV (Wang and Sang, 2018). It has been observed in different berries (Rimando et al., 2004) and red wine (Pezet et al., 1994). It was reported that, due to its lipophilic structural characteristic, pterostilbene exhibits better bioavailability than RV (Kapetanovic et al., 2011). Pterostilbene metabolism encompasses glucuronidation and sulfatation, being this last predominant (Kapetanovic et al., 2011). Shao et al. (2010) also identified other metabolites in mice urine such as mono-hydroxylated and demethylated pterostilbene derivatives (Shao et al., 2010). In addition, pinostilbene (a demethylated pterostilbene) was recently identified as a mayor pterostilbene colonic metabolite in mice (Sun et al., 2016). However, no pharmacokinetic investigations of pterostilbene have been performed in humans yet. Therefore, complementary studies are also necessary for a better understanding of its metabolism and properties.

Resveratrol has been the most widely stilbene studied and a great number of activities have been endorsed, including neuroprotective capacity (Fernández-Mar et al., 2012). Recently, it has been proved that RV and its major metabolites crossed the human BBB, showing CNS effects, with their measurable amounts detected in plasma and cerebrospinal fluid (CSF) (Turner et al., 2015). This fact highlights that this compound could be taken in consideration as a neuroprotective molecule.

Nowadays, the study of the health effects of other stilbenes is gaining importance due to the existing evidence proving more potent activity for RV derivatives or related compounds than RV (Zghonda et al., 2011, 2012).

## RV and Other Stilbenes and the Molecular Mechanisms Implicated in Their Anti-Inflammatory Activities

Regarding the anti-inflammatory effects of stilbenes (RV and derivatives), an interesting number of works have been published being the *in vitro* studies more extensive than *in vivo* ones. In this review, a total of 23 *in vitro* and 6 *in vivo* works have been selected (Table 1).

**Table 1:** Summary of RV and derivatives activities (*in vivo* and *in vitro*) in counteracting neuroinflammation.

Model	Compounds	Dose	Microglia activated by	Effect	Reference
***In vivo***										
Adult male C57/BL6 mice	RV	20 mg/kg (14 consecutive days)	LPS	Reduction of microglia activation (Iba-1 + cells) Inhibition of the NF-κB in the hippocampus Induction of activation of SIRT1	Liu et al., 2016
Male Sprague–Dawley rats	Pterostilbene	20 mg/kg (3 days)	LPS	Mitigation of microglial activation in rat hippocampal CA1 and dentate gyrus (DG) Inhibition of IL-6 and TNF-α mRNA expression in rat hippocampus and rat serum	Hou et al., 2015
APP/PS1 transgenic mice	RV ALD55: (E)-2-fluoro-4 -methoxystilbene)	100 ppm (0.01% weight) (1 year)	Aβ	Reduction of microglia activation (Iba-1 + cells) Reduction of Aβ plaque density	Solberg et al., 2014
Male adult Wistar rats	RV (Free and in lipid core nanocapsule)	10 mg/kg/day (14 consecutive days)	Aβ_1-42_	Reduction of astrocyte and microglial activation and block JNK activation (RV in lipid core nanocapsule) Increase in phosphorylation/inactivation of GSK-3β (RV and RV in lipid core nanocapsule)	Frozza et al., 2013
APP/PS1 transgenic mice	RV	Diet supplementation (0.35% of RV) (15 weeks)	Aβ	Reduction of microglial activation (Iba-1 + cells)	Capiralla et al., 2012
BALB/c aged mice	RV	Diet supplementation (0.4% of RV) (4 weeks)	LPS	Reduction of IL-1β in plasma and in hippocampus Improvement the impaired spatial working memory	Abraham and Johnson, 2009
***In vitro***					
C8-B4 microglial cells	RV	100 μM	LPS and IFN-γ	Reduction of NO, IL-1α, IL-1β,IL-6, and TNF-α	Steiner et al., 2016
BV-2 microglial cells	RV	0–30 μM	Oligomeric Aβ (oAβ)	Inhibition of ROS, NO, TNF-α, and IL-1β	Yao et al., 2015
				Inhibition of protein expression levels of p47^phox^ and gp91^phox^ (NADPH oxidase)	
N13 microglial cells	RV		LPS		Cianciulli et al., 2015
		1–20 μM		Reduction of IL-1β, TNF-α and IL-6 mRNA expression	
				Increment of IL-10	
				Increase of JAK1^phox^ and STAT3^phox^ and suppression of SOCS3 (JAK–STAT signaling pathway)	
Primary microglia	RV	1 μM	LPS	Inhibition of microglial activation	Wang et al., 2015
				Reduction of IL-1β, iNOS, COX-2 and TNF-α	
				Blockage NF-κB stimulation	
BV-2 microglial cells	Pterostilbene	1–30 μM	LPS	Suppression of NO, iNOS, IL-6, and TNF-α mRNA expression	Hou et al., 2015
				Inhibition of the phosphorylation of MAPKs	
N13 microglial cells	RV	10 μM	LPS	Modulation of SOCS-1 dependent PI3K/Akt signaling cascade	Dragone et al., 2014
				Reduction of ROS, SOD, p38, PI3K/Akt, NF-κB activation, NO, and iNOS	
Primary microglia, astrocytes, and mixed glial cell cultures	RV	5, 10, and 20 μM		Reduction of MPO, NO, IL-1β, COX-2, TNF-α, and iNOS	Chang et al., 2013
			Rotenone	Reduction of gp91^phox^ (NADPH oxidase)	
BV-2 microglial cells				Attenuation of cell death in co-culture	
Rat primary cortical neuron-glia cultures	RV	15–60 μM	LPS	Inhibition of microglial activation (decrease of Iba-1 + cells)	Zhang et al., 2013
				Inhibition of TNF-α, iNOS, IL-1β, and NO	
BV-2 microglial cells	RV	50 μM	LPS	Inhibition of NF-κB activation (interfering with IKK and IκB phosphorylation)	Capiralla et al., 2012
			Aβ	Reduction of IL-6, TNF-α, M-CSF, MCP-1, MCP-5, CD54, IL-1ra, IL-27, iNOS, and COX-2	
				Diminution of STAT1 and STAT3	
				Reduction of FLAG-tagged TLR4	
BV-2 microglial cells	RV	25–100 μM	LPS	Attenuation of NO, PGE2, iNOS, COX-2,TNF-α, IL-1β, and NF-κB	Zhong et al., 2012
N9 microglial cells	RV	0–50 μM	LPS	Inhibition of TNF-α, IL-6, MCP-1, IL-1β, and iNOS/NO	Lu et al., 2010
Primary microglia				Inhibition on LPS-stimulated phosphorylation of JNK in (astrocytes)	
Primary astrocytes				Inhibition of NF-κB activation (inhibition of AP-1 activation only in microglia)	
Primary rat midbrain neuron-glia and neuron-astroglia cultures	RV	60 μM	LPS	Reduction of NADPH oxidase-mediated generation of ROS	Zhang et al., 2010	
				Attenuation of translocation of NADPH p47	
				Implication of MAPK and NFκB signaling pathways	
Microglial BV-2 cells	RV	0–50 μM	LPS	Inhibition of IL-1β production	Abraham and Johnson, 2009
N9 microglial cell line	RV	0.3–30 μM	LPS	Suppression NO and ROS production	Meng et al., 2008c
Cultured rat cortical microglia				Inhibition of iNOS	
				Attenuation of TNF-α	
				Blockage of IκBα phosphorylation and degradation	
Primary rat microglia		0–30 μM	LPS	Reduction of iNOS	Meng et al., 2008b
	21 RV derivatives			Inhibition of TNF-α by blocking IκBα phosphorylation and degradation	
	RV	0.1 μM	LPS		Bureau et al., 2008
N9 microglial cells				Reduction IL-1α and TNF-α	
N9 microglial cells		0–30 μM	LPS		Meng et al., 2008a
Primary rat microglial cells	RV			Inhibition of NO production and iNOS expression	
Primary microglial cell cultures	RV	1–50 μM	LPS	Reduction of PGE2 synthesis and formation of 8-iso-PGF2α and mPGES-1	Candelario-Jalil et al., 2007
				Inhibition of COX-1	
C6-microglial cells	RV	0.5–20 μM	LPS	Inhibition of NO, PGE2, iNOS, and COX-2	Kim et al., 2007
				Suppression of translocation and activation of NF-κB	
BV-2 microglial cells	75 *trans*-stilbenes		LPS	Inhibition the TNFα-induced activation of NFκB	Heynekamp et al., 2006
				Diminution of COX-2 mRNA expression	
BV-2 microglial cells	Piceatannol	0–40 μM	LPS	Inhibition of the release of NO, PGE2, IL-1β, IL-6, TNF-α, iNOS and COX-2	Jin et al., 2006
				Prevention of NF-κB p65 nuclear translocation	
Cultured rat cortical microglia and N9 microglial cells	RV	0.01, 0.1, 1, and 10 μg/mL	LPS	Inhibition on the production of TNF-α, NO, iNOS	Bi et al., 2005
				Suppression of degradation of IκBα	
				Reduction of phosphorylation of p38 (MAPKs signaling pathway)	


Regardless of the different clinical and pathological features between AD and PD, ultimately leading to neuronal cell death, they share common molecular mechanisms such as protein aggregation, oxidative stress, mitochondrial dysfunction and neuroinflammation (Irvine et al., 2008; Yan et al., 2013).

With regard to stilbenes, it may be interesting to underline that several properties have been described including neuroprotective activities at different levels such as anti-amyloidogenic efficacy, neuroprotection via modulation of neural mediators and enzymes and interaction/modulation with different signaling pathways (Basli et al., 2012). For example, it has been demonstrated that they can act by reducing the intracellular Aβ levels and by inhibiting Aβ fibril formation and toxicity *in vitro* (Rivière et al., 2007, 2010; Temsamani et al., 2016). Moreover, stilbenes have demonstrated to be effective free-radical scavengers protecting against oxidative stress through the activation of nuclear factor-erythroid-2-related factor-2 (Nrf2) and sirtuin 1 (SIRT1) pathways (Pallàs et al., 2009; Reinisalo et al., 2015).

Additionally, oxyresveratrol (the hydroxylated derivative of RV) has shown neuroprotective effects against 6-OHDA, a catecholaminergic neurotoxin formed in PD patients, acting via the reduction of intracellular reactive oxygen species (ROS), attenuation of phospho-c-Jun N-terminal kinase (JNK)-1 and phospho-JNK-2 and increase in cytosolic SIRT1 levels (Chao et al., 2008). Furthermore, Amurensin G (a RV dimer) enhances cell viability in SH-SY5Y cells and inhibits rotenone-induced cell cycle arrest by decreasing G2/M involving an autophagic activity (Ryu et al., 2013). Similarly, amurensin G is reported to protect against Aβ_(25-35)_-mediated neurotoxicity in rat cerebral cortical neurons and in mice (Jeong et al., 2010).

In general, several mechanisms of RV and its derivatives have been proposed on microglia-mediated neuroinflammation including: Nuclear Factor-Kappa B (NF-κB), MAPKs, Janus Kinase/Signal Transducer and Activator of Transcription (JAT/STAT) and SIRT1 pathways.

### NF-κB Pathway

NF-κB is an important transcription factor responsible of the regulation and production of pro-inflammatory factors, including NO, TNF-α, and IL1-β (Lawrence, 2009). NF-κB is normally located in the cytoplasm by binding of its inhibitors IκBs. IκBs are rapidly phosphorylated and degraded via IKK complex when an inflammatory insult occurs, leading to the liberation of NF-κB dimers (p50 and p65). Then, these dimers are translocated to the nucleus regulating the expression of numerous target genes (TNF-α, iNOS, IL-1β, and IL-6 among others) (Tak and Firestein, 2001; Rahman and Fazal, 2011).

The effect of stilbenes to prevent the nuclear translocation of NF-κB and the consequent liberation of pro-inflammatory cytokines is one of the well-known and most studied pathways. Multiple works have revealed that RV and it analogs and other stilbenes such as piceatannol are able to prevent the liberation of pro-inflammatory cytokines acting by inhibiting the NF-κB transcription and expression.

Therefore, RV (0.04–43.8 μM) suppresses the degradation of IκBα in LPS-stimulated N9 microglial cells and as result of this, inhibits the iNOS expression (Bi et al., 2005). In accordance with this paper, other authors showed that RV at low concentrations (0.5–20 μM) markedly inhibited LPS-mediated nuclear translocation of NF-κB protein and transcriptional activation of NF-κB promoter in C6- microglial cells (Young et al., 2007). Moreover, using microglial and astrocytes cell lines it has been described that RV (5, 25, and 50 μM) can suppress the NFκB activation in both types of cells and also inhibits the AP-1 in microglia (Lu et al., 2010). AP-1 also acts by activating the extracellular signal-regulated kinase (ERK) subgroup of MAPKs (Fujioka et al., 2004) being this another interesting pathway to combat neuroinflammation.

Additionally, it has been demonstrated that after stimulation of microglia either using LPS or fibrillary Aβ, RV (50 μM) inhibits the NF-κB activation upon LPS stimulation by interfering with IKK and IκB phosphorylation. As a consequence of this inhibition, they (Capiralla et al., 2012) also observed a reduction of the gene expression of IL-6, M-CSF, MCP-1, MCP-5, CD54, IL-1ra, IL-27. Furthermore, RV was able to inhibit the fibrillar Aβ-triggered increase of STAT1, STAT3, and IκBα phosphorylation and also the TNF-α and IL-6 secretion. Moreover, they also demonstrated using a transgenic mice model of AD (APP/PS1), that the supplementation of the diet with 0.35% of RV resulted in a reduction of microglial activation, observing a decrease on the number of Iba-1 cells (Capiralla et al., 2012). The blocking of NF-κB activation by RV (1 μM) has been also recently observed with primary microglia cultures after LPS-stimulation with the confirmation of the reduction of IL-1β, iNOS, COX-2, and TNF-α levels and in consequence, the protection of primary hippocampal neurons (Wang et al., 2015).

Piceatannol (20 and 40 μM) another widely known stilbene compound present in wine, has demonstrated its capacity to prevent the NF-κB p65 nuclear translocation as well as the inhibition of the release of NO, PGE-2, the inhibition of the transcription of IL-1β, IL-6 and TNF-α and the attenuation of the expression of iNOS and COX-2 mRNA and protein levels in LPS treated BV2 microglial cells (Jin et al., 2006).

Furthermore, MAPKs are a highly conserved family of serine/threonine protein kinases involved in a great variety of cellular processes such as proliferation, differentiation, motility, stress response, apoptosis and survival (Mordret, 1993; L’Allemain, 1994). Extracellular ERK1/2, JNK, and p38 are the three principal components of MAPK (Cargnello and Roux, 2011; Arthur and Ley, 2013). When extracellular pathogenic and noxious stimuli induce inflammation, MAPKs are activated and translocate to the nucleus where the activation of the transcription machinery of pro-inflammatory genes giving rise to the increase of TNF-α and iNOS takes place. In addition, MAPK participates in the regulation of NF-κB transcriptional activity, thus JNK and p38 are implicated on the cytoplasmatic and nuclear NF-κB activation (Schulze-Osthoff et al., 1997).

Some *in vitro* studies have proved that RV is a potent inhibitor of the phosphorylation of p38, ERK1/2 and JNK induced by LPS in microglial cells (Bi et al., 2005; Zhang et al., 2010; Dragone et al., 2014) and in astrocytes (Lu et al., 2010). Additionally, using lipid-core nanocapsules as a RV carrier, they observed that higher intracerebral concentrations of RV were achieved in those rats injected with Aβ_1-42_, this fact related with the neuroprotective effect observed. This work also reported the blockage of JNK as a mechanism associated with the protection of astrocyte and microglial activation and Aβ triggering cell disruptions (Frozza et al., 2013).

Furthermore, pterostilbene (1–30 μM) significantly suppresses LPS-induced NO production and iNOS mRNA expression, IL-6 and TNF-α production and mRNA expression and phosphorylation of MAPKs (p38, JNK, and ERK) in BV-2 microglial cells, which also demonstrates that this pathway is involved in the observed effect. In addition, *in vivo* data also showed a significantly inhibition of LPS-induced IL-6 and TNF-α mRNA expression in rat hippocampus and a reduction of their amount in rat serum (Hou et al., 2015).

### NADPH Oxidase Pathway

NADPH oxidase is recognized as the key ROS-producing enzyme during inflammation together with iNOS, and is widely expressed in various immune cells including macrophages and microglia (Hernandes and Britto, 2012). This enzyme is required for the production of ROS in activated microglia. Once NADPH oxidase is activated, the cytosolic subunits (p40^phox^, p47^phox^, p67^phox^, and Rac1) are translocated to the membrane-binding cytochrome b558 which consists on the union of p22^phox^ and gp91^phox^ forming the functional oxidase that catalyzes the reduction of oxygen to superoxide free radical (Infanger et al., 2006). Several studies have indicated that both pharmacological inhibition and/or the genetic deletion of NADPH oxidase protects against LPS, rotenone, paraquat, and 1-methyl-4-phenyl-1,2,3,6-tetrahydropyridine (MPTP)-induced neurodegeneration (Gao et al., 2011). Is for this reason that NADPH oxidase pathway can represent a potential target for neuroinflammation-related neurological disorders.

Various articles have been published regarding the role of RV and other stilbenes on NADPH-oxidase pathway. An *in vitro* study has shown that RV (3, 10, and 30 μM) protects against oligomeric Aβ-induced microglial activation by inhibiting the expression of NADPH oxidase, and that both gp91^phox^ and p47^phox^ subunits were involved in this reaction (Yao et al., 2015). These results are in accordance with another study in which primary microglia was activated with rotenone, a pesticide that causes a systemic defect in mitochondrial complex I and oxidative stress, contributing to the pathogenesis of PD (Betarbet et al., 2000). The authors found that RV (10 μM) reduced the gp91^phox^ levels. Moreover, RV (5, 10, and 20 μM) noticeably suppressed the rotenone-induced expression of a pool of pro-inflammatory mediators, including TNF-α, COX-2, and iNOS and reduced the NO and myeloperoxidase (MPO) [oxidant-generating enzyme that catalyzes the formation of the potent oxidant hypochlorous acid (HOCl) and other chlorinating species derived from H_2_O_2_ levels] (Chang et al., 2013).

### SIRT 1 and AMPK Pathway

Another pathway to take into consideration, due to its implication in neuroinflammation, is SIRT1/AMPK that is recognized as a longevity-regulating pathway. SIRT1 is an enzyme of the sirtuin class of nicotinamide NAD^+^-dependent histone deacetylases, which has been implicated in a wide range of biological processes including cell survival, metabolism, DNA repair and aging and that is deemed to be a nuclear sensor of redox signaling (North and Verdin, 2004). In addition, SIRT1 acts by inactivating NF-κB by deacetylating the RelA/P65 subunit at lysine 310 (Howitz et al., 2003; Yeung et al., 2004). For this reason, this signaling pathway plays an important role in inflammation and can serve as a potential target to treat inflammation-related disorders (Salminen et al., 2013). A close relation between SIRT1 and AMPK pathways has been described. In fact, RV has demonstrated to increase the lifespan in a SIRT dependent manner *in vivo*, leading to AMPK activation via deacetylation and activation of the AMPK kinase LKB1 (Price et al., 2012).

Only one study (*in vivo*) has reported that RV (20 mg/kg intraperitoneal injection during 14 consecutive days) induced the activation of SIRT1 reversing LPS-induced depression-like behaviors by enhancing neurogenesis in C57/BL6 mice. In this study authors observed a reduction of microglia activation (Iba-1 cells) and moreover, an inhibition of the LPS-induced increase of NF-κB in mice hippocampus (Liu et al., 2016).

### Suppressor of Cytokine Signaling (SOCS) and JAK-STAT Pathway

Suppressor of cytokine signaling proteins are a family of eight members expressed by immune cells and the CNS cells that regulate immune processes, including microglia activation (Campbell, 2005).

The expression of SOCS-1 is initially controlled by STAT1 and STAT3 activation, but their expression can be also arbitrated by MAPK and NF-κB signaling cascades (Shuai and Liu, 2003; Croker et al., 2008).

Moreover, the JAK–STAT signaling pathway is an important signal transduction cascade and it is critical for the regulation of almost 40 cytokine receptors signal (Murray, 2007). STAT3, when is phosphorylated by the receptor-associated JAKs, translocate to the nucleus where it binds with a high affinity to the promoters of various genes. SOCS3 is one of these gens and operates as a negative regulator of cytokine-induced responses and, consequently suppressing pro-inflammatory cytokine activity (Starr et al., 1997).

The link between this pathway and the anti-inflammatory properties of RV has also been studied. Thus, RV pretreatment at low concentrations (1–20 μM) has shown to be able to significantly up-regulate the phosphorylated forms of JAK1 and STAT3, as well as increase the cytokine signaling SOCS-3 protein expression in LPS activated microglial cells, demonstrating the capacity of RV to modulate the JAK–STAT signaling pathway (Cianciulli et al., 2015). These results are also in accordance with other work that also proved that RV (50 μM) acted via a mechanism involving Akt/NF-κB/STAT signaling pathway and least in part due to the RV capacity of inhibit the Toll Like Receptor 4 (TLR4) oligomerization (Capiralla et al., 2012).

Furthermore, Dragone et al. (2014) noted for the first time that RV (10 μM) is able to induce SOCS-1 expression both in un-stimulated and in LPS-stimulated murine N13 microglial cells, suggesting that it may play an important neuroprotective role, by reducing microglia activation. This conclusion was also supported by the reduction of superoxide anion and NO production, the reduction on levels of TNF-α, IL-1β, and IL6 as well as the reduction of p38, PI3K/Akt and iNOS expression.

### Glycogen Synthase Kinase-3 (GSK-3)

Glycogen synthase kinase-3 is a multifunctional serine/threonine kinase found in all eukaryotes and it is involved in multiple cellular processes, including neurogenesis, motility and survival (Doble and Woodgett, 2003). In addition, GSK-3 has been reported as an important regulator of microglia promoting migration and a promotor of the production of inflammatory cytokines, and the inflammation-induced neurotoxicity. It has been demonstrated that the inhibition of GSK-3 attenuates by 70% LPS-induced IL-6 production and by 80% the NO production (Yuskaitis and Jope, 2009). Furthermore, GSK-3 regulates selectively the expression of CD11b, a marker of microglial activation. Thus, GSK-3 can directly lead to the CD11b expression either by regulating transcription factors, such as NF-κB or by inducing the production of inflammatory mediators that can induce CD11b expression, such as IL-6, TNF-α and NO (Roy et al., 2006). Additionally, regarding AD, a selective GSK-3 inhibitor tested in transgenic mouse model has been shown to have capacity to reduce Aβ levels by clearance and neuroinflammation (Licht-Murava et al., 2016).

In this context, one work has reported that RV (free RV and RV-loaded lipicore nanocapsules) (5 mg/kg/day, each 12 h) produced a noticeable inactivation of GSK-3β in injected Aβ_1-42_ rats (Frozza et al., 2013).

### IL-10

IL-10 is an immunoregulatory and anti-inflammatory cytokine that is able to inhibit the production of pro-inflammatory cytokines after LPS insult (Ledeboer et al., 2000; Molina-Holgado et al., 2001). Additionally and in relation with the above mentioned, IL-10 expression is well known to be dependent on the JAK/STAT signaling pathway by activating STAT3, which is mainly involved in the negative regulation of macrophage activation (Moore et al., 2001).

Recently, RV (10 μM) has demonstrated to be effective increasing in a dose dependent manner, both mRNA and protein IL-10 levels and decreasing the pro-inflammatory cytokines IL-1β, TNF-α, and IL-6 mRNA expression. In this study authors also showed that RV pretreatment up-regulated the phosphorylated forms of JAK1 and STAT3, as well as SOCS3 protein expression in LPS activated cells (N13 microglial cells) (Cianciulli et al., 2015).

## OLE and HT: Bioavailability, Pharmacokinetics and Blood–Brain Barrier (BBB) Permeability

Virgin olive oil is the main fat source in MD and within its minor components polyphenols play a significant role. There are more than 100 different biophenols reported in olive samples, being the major, HT, tyrosol and their secoiridoid derivatives (OLE, OLE aglycone and elenolic acid dialdehydes) (El Riachy et al., 2011; Obied et al., 2012). OLE, HT and tyrosol are the main polyphenols present in EVOO and extensive research has been conducted regarding their bioactivity, mainly related with cardiovascular protection. More recently, they are the focus of studies in the field of neuroprotection (Rodríguez-Morató et al., 2015; Martínez-Huélamo et al., 2017). HT is a product of the hydrolysis of OLE, formed during the maturation and storage of olive oil, and the preparation of table olives (Vissers et al., 2002). OLE is an ester of HT and the elenolic acid glucoside (Bendini et al., 2007). During olive fruit processing glycosides are hydrolysed by endogenous β-glucosidases. HT is the major component of the polyphenol fraction in olive oil, its content ranging from 50 to 200 mg/kg oil for EVOO (Visioli and Bernardini, 2011; Romero and Brenes, 2012). Noteworthy the concentration of polyphenols in VOO is affected by many different factors such as olive cultivar, geographical area, age of the tree, agronomic and environmental factors, degree of ripeness as well as by the extraction system and storage conditions (Servili et al., 2004; Martín-Peláez et al., 2013).

Hydroxytyrosol derived from its natural sources is bioavailable for humans, being metabolized and excreted in urine as glucuronide and sulfate derivatives (Visioli et al., 2000; EFSA Panel on Dietetic Products et al., 2017). The degree of absorption is outstanding being higher than 40% for HT (Visioli et al., 2001; Tuck and Hayball, 2002; Vissers et al., 2002). Being HT a polar compound, its absorption takes place by passive transport in the small bowel and the colon (Manna et al., 2000). HT is more assimilated when given as EVOO compared to an aqueous solution due to the protection of antioxidants (Tuck et al., 2001). Moreover, its absorption was greater when the intake was as EVOO rather than added in refined olive oil or into a yogurt (Visioli et al., 2003). These results show how the antioxidants present in EVOO protect HT from degradation in the gastrointestinal tract. HT precursors, OLE and OLE aglycon, also known as secoiridoids are less polar and they may be rapidly hydrolyzed yielding HT (Vissers et al., 2004; Corona et al., 2006). Therefore, attention on the biological effects is mainly focused on HT. On the other hand, OLE, as a glycoside molecule, may reach the colon unaltered generating more diverse microbial metabolites (López de las Hazas et al., 2016). Nevertheless certain studies refer that OLE can be readily absorbed across the intestine (Edgecombe et al., 2000) by possible implication of glucose transporter. Further research is required to substantiate the mechanisms of absorption for these phenolics (Cicerale et al., 2010).

Subsequently, extensive metabolism takes place first in the gut and subsequently in the liver. Gut microflora acts transforming part of HT into hydroxylated phenylacetic acids (Mosele et al., 2014). The enzymes involved in HT phase-II reactions in the liver are sulfotransferases and uridine 5′-diphosphoglucuronosyl transferases (UGTs), resulting in the correspondent HT metabolites detected in biological samples. Also acyltransferases are able to form HT acetate (Rubió et al., 2012). Moreover, D’Angelo et al. (2001) demonstrated also that HT undergoes enzymatic oxidation and methylation processes driven the formation of 3,4-di-hydroxyphenylacetaldehyde and subsequently 3,4-dihydroxyphenylacetic acid (by the alcohol and aldehyde dehydrogenases), and 4-hydroxy-3-methoxyphenylethanol also called homovanillic acid (by the catechol ortho methyl transferase). All these compounds are transformed into sulfo conjugates by a sulfotransferase enzyme (Robles-Almazán et al., 2018). HT sulfate is the main circulating metabolite detected in rat plasma (D’Angelo et al., 2001; Serra et al., 2012), whereas in humans, HT-sulfate together with HT acetate sulfate are the main metabolites detected in plasma after the consumption of HT or HT derivatives at normal dietary doses (Mateos et al., 2011; Rubió et al., 2012). Additionally, free HT, ortho-methyl products of HT (homovanillic alcohol and acid), glucuronide derivatives and glutathionyl conjugates can be also found in plasma (Rodríguez-Morató et al., 2016). HT and their metabolites may be also redirected to the biliary excretion route; hence the enterohepatic recycling would enable a longer exposure of HT and metabolites (Serra et al., 2012). Therefore, not only HT but also its metabolites should account for its health benefits. Besides, it has been recently pointed that HT metabolism depend on the gender, being females more efficient in the transformation and utilization of HT (Domínguez-Perles et al., 2017).

Furthermore, HT is also present in wines and urinary recoveries of HT were higher than expected after red wine administration, probably due to the interaction between ethanol and dopaminergic pathways (de la Torre et al., 2006). HT is a known dopamine metabolite and hence (if the intake includes ethanol), dopamine metabolism turns to produce HT instead of DOPAC (3,4-dihydroxyphenylacetic acid) (Boileau et al., 2003; Perez-Mana et al., 2015). HT is present in the brain since it is a dopamine metabolite (de la Torre et al., 2006; Mosele et al., 2014). Deamination of dopamine by monoaminoxidase yields DOPAL (3,4-dihydroxyphenylaldehyde), that can be oxidized by aldehyde dehydrogenase to DOPAC. In a lesser extent, DOPAL may be reduced to HT by the ALR and HT can be converted to DOPAL by means of ADH. At the same time DOPAC can be transformed into HT by DOPAC reductase (Xu and Sim, 1995).

Hydroxytyrosol is closely related to cardiovascular protection and blood lipid stabilization since once absorbed into the blood stream, it will be joined to plasmatic low-density lipoproteins, acting as an antioxidant (EFSA Panel on Dietetic Products, Nutrition and Allergies [NDA], 2011; Fernández-Ávila et al., 2015). Due to the fact of the rapid metabolism its plasma half-life is estimated in 1–2 min (D’Angelo et al., 2001; Granados-Principal et al., 2014). The metabolites reach different organs and tissues and even the brain, so they comply with the requirement of crossing the BBB to be used as a neuroprotective agents (D’Angelo et al., 2001). The content of HT in rat brain has been the subject of extensive research, reporting basal HT contents at very low levels of several units ng/g (Wu et al., 2009; Serra et al., 2012; Gallardo et al., 2014; Goldstein et al., 2016; Peng et al., 2016).

Summarizing, exposure to HT results not only from the intake of free HT, but to a significant degree also from ingested OLE and its aglycone contained in olives and EVOO. HT derived from its natural sources is bioavailable for humans, metabolized and rapidly eliminated primarily in the urine as glucuronide and sulfate derivatives.

## OLE, and HT and the Molecular Mechanisms Implicated in Their Anti-Inflammatory Activities

A large body of evidence from clinical trials and population studies indicates that olive phenolic compounds are key responsible for the MD protective effects against aging-associated cognitive impairment and neurodegenerative diseases such AD and PD, as well as for the improvement of cognitive performance (Di Giovanni, 2009; Scarmeas et al., 2009; Alcalay et al., 2012; Gardener et al., 2012; Casamenti et al., 2015; Safouris et al., 2015; Peyrol et al., 2017; Robles-Almazán et al., 2018).

Oleuropein and HT have shown neuroprotective activity by acting against oxidation and inflammation and interfering with amyloid Aβ and tau protein aggregation. Hence, HT, OLE, and OLE aglycon may counteract ROS formation and avoid the amyloid plaque generation and deposition (Daccache et al., 2011; Rigacci et al., 2011; Barbaro et al., 2014; Rigacci and Stefani, 2015), critical processes in the initiation of AD pathology. In addition, oleocanthal (0.01–10 μM) reported its ability to interact with Aβ aggregation, providing neuroprotective benefits on primary hippocampal cultures (Pitt et al., 2009). Moreover, OLE aglycone oral administration (12.5 mg/kg of diet) also improved cognitive deficits and reduced Aβ42 plaque area and number and induced autophagosome-lysosome system in the cortex of a transgenic AD mouse model (Pantano et al., 2017).

Transgenic *Caenorhabditis elegans* strains expressing Aβ42 has been used as a model of invertebrate AD (Link, 2005). OLE was added to the grown medium and it was able to interfere with the Aβ aggregation avoiding the appearance of toxic species (Diomede et al., 2013). In addition, Peng et al. (2016) reported that HT reduces brain mitochondrial oxidative stress and neuroinflammation in AD-prone transgenic mice by induction of Nrf2-dependent gene expression. These recent findings suggest that HT, thanks to its ability to restore homeostasis and induce appropriate stress response pathways (hormesis) could be considered a potential therapeutic target in neurodegenerative diseases opening new prospective in the field of neuroprotection.

Specifically in microglia, we have found three works (2 *in viv*o and 1 *in vitro*) related with the effects of OLE at this level (Table 2). The oral administration of OLE aglycone (450 μM) found in olive leaves, significantly attenuated astrocyte and microglial activation in an Aβ42-induced AD rat model by interfering with Aβ aggregation (Luccarini et al., 2014). In addition, dietary supplementation of OLE aglycone on young/middle-aged TgCRND8 mice (50 mg/kg; 8 weeks) reduced Aβ levels and plaque deposits and produced the microglia migration to the plaques. Moreover, OLE demonstrated to strongly promotes a phagocytic response and lysosomal activity (Grossi et al., 2013). Data obtained with cultured cells (BV-2 microglial cells) showed the capacity of OLE (1, 5, and 10 μM) to inhibit the production of pro-inflammatory cytokines via regulation of ERK, P38 (MAPKs) and NF-κB activation. This work has also demonstrated that OLE can affects the LPS-induced mitochondria fission acting by decreasing the number of fragmented and elongated mitochondria via dephosphorylation of the Drp1 (Park et al., 2017).

**Table 2:** Summary of OLE activities (*in vivo* and *in vitro*) in counteracting neuroinflammation.

Model	Compound	Dose	Microglia activated by	Effect	Reference
Male Wistar rats	OLE	450 μM	Aβ_1-42_	Attenuation of astrocytes and microglia reaction	Luccarini et al., 2014
Transgenic CRND8 mice	OLE	50 mg/kg of diet (8 weeks)	Aβ	Diminution of astrocyte reaction Increase of microglia migration (phagocytosis of amyloid deposits)	Grossi et al., 2013
BV-2 microglial cells	OLE	1, 5, and 10 μM	LPS	Suppression of NO (via ERK/p38/NF-κB activation) and ROS generation Suppression of mitochondrial fission (regulates mitochondrial ROS generation and pro-inflammatory response by diminishing Drp1 dephosphorylation	Park et al., 2017


Concerning HT, some articles have been published in macrophages cell lines. The first study reported by Maiuri et al. (2005) proved that HT (at high concentration; 200 μM) inhibits iNOS and COX-2 expression in LPS-stimulated J774 cells by preventing the activation of NF-κB, STAT-1α, and IRF-1. Moreover, others authors reported that HT inhibited the production of NO and PGE2 with an IC_50_ of 11.4 and 19.5 μM, respectively (much lower concentrations) in LPS-stimulated RAW 264.7 cells. Additionally, they also notified a diminution on the cytokines secretion (IL-1α, IL-1β, IL-6, IL-12, and TNF-α) and chemokines (CXCL10/IP-10 and CCL2/MCP-1) acting also via NF-κB pathway (Richard et al., 2011). Similar results were obtained by Takeda et al. (2014). Other interesting work using nutritional relevant concentrations of HT and OLE (50 and 10 μM) demonstrated that HT (10 μM) inhibits the production of NO and PGE2 and that is also able to induced de Nrf2 nuclear translocation in LPS treated RAW 264.7 (Bigagli et al., 2017). The Nrf2 is considered a master regulator of redox homeostasis but its activation also inhibit proinflammatory mediators including cytokines, COX-2 and iNOS (Ahmed et al., 2017).

Although macrophages and microglia share similar features regarding their morphology and functions, the polarization pattern in microglial cells is much more complex than that observed in macrophages. Therefore, the study of the anti-neuroinflammatory activity of HT in microglial cells lines remain nowadays unexplored, being an interesting line of research that will be take in consideration for the scientific community.

## Conclusion

Moderate intake of red wine and EVOO are distinctive features of the MD. Both food items are rich source of polyphenolic compounds, such as RV and HT and their derivatives with demonstrated neuroprotective properties including anti-inflammatory effects on microglia. This fact makes them possible candidates for co-adjuvant therapies to treat neurodegenerative diseases such as AD and PD prevention.

New strategies that may induce a shift in microglial cells from inflammatory and neurotoxic phenotype to an anti-inflammatory and neuroprotective one is currently an objective of the scientific community. In this sense, several mechanisms have been proposed for the anti-inflammatory and neuroprotective effect of stilbenes and HT and its derivatives. Thus, stilbenes acts: (i) preventing the nuclear translocation of NF-κB, reducing the production of pro-inflammatory factors IL-1β, iNOS, COX-2, and TNF-α levels; (ii) inhibiting the expression of NADPH oxidase, (iii) inducing the activation of SIRT1/AMPK which reduce microglia activation; (iv) suppressing the cytokine signaling SOCS and JAK-STAT pathway; and (v) increasing both mRNA and protein levels of the anti-inflammatory cytokine IL-10. On the other hand, OLE significantly attenuates microglial activation acting via NF-κB activation. However, further research on anti-neuroinflammatory effect of HT in microglial is needed.

Nowadays, the study of the neuroprotective effects of other stilbenes as well as HT derivates present on the MD are gaining importance and represents an important new via of research since derivatives or related compounds might display more potent activity than the pair one.

## Author Contributions

RH-O and AC literature search and first draft. RdP, SK, TR, AT, and MG-P thorough revision and discussion and final document.

## Conflict of Interest Statement

The authors declare that the research was conducted in the absence of any commercial or financial relationships that could be construed as a potential conflict of interest.

## Abbreviations

α-syn: α-synuclein
6-OHDA: 6-hydroxydopamine
Aβ: amyloid-β
AD: Alzheimer’s disease
ADH: alcohol dehydrogenase
ALR: aldehyde/aldose reductase
AMPK: adenosine monophosphate-activated protein kinase
AP-1: activator protein -1
BBB: blood-brain barrier
CNS: central nervous system
COX-2: cyclooxygenase 2
DG: dentate gyrus
DOPAC: 3,4-dihydroxyphenylacetic acid
DOPAL: 3,4-duhydroxyphenyl aldehyde
Drp1: dynamin-related protein 1
EFSA: European Food Safety Authority
ERK: extracellular signal-regulated kinase
EVVO: extra virgin olive oil
GSK-3: glycogen synthase kinase-3
HT: hydroxytyrosol
IL: interleukin
INF-γ: interferon-γ
iNOS: inducible nitric oxide synthase
JAK: Janus kinase
JNK: c-Jun N-terminal kinase
LPS: lipopolysaccharide
MAPKs: mitogen-activated protein kinases
MD: Mediterranean diet
MPO: myeloperoxidase
MPTP: 1-methyl-4-phenyl-1,2,3,6-tetrahydropyridine
mTOR: mammalian target of rapamycin
NAD: adenine dinucleotide
NADPH: nicotinamide adenine dinucleotide phosphate hydrogen
NF-κB: nuclear factor-Kappa B
NO: nitric oxide
Nrf2: nuclear factor–erythroid 2-related factor
oAβ: oligomeric Aβ
OLE: oleuropein
PD: Parkinson’s disease
PGE-2: prostaglandin E2
PI3K/Akt: phosphatidylinositol-3-kinase and protein kinase B
ROS: reactive oxygen species
RV: resveratrol
SIRT: sirtuin
SN: substantia nigra
SOCS: suppressor of cytokine signaling
STAT: signal transducer and activator of transcription
STS: stilbene synthase
TGB-β: transforming growth factor beta
TNF-α: tumor necrosis factor
UGTs: uridine 5′-diphosphoglucuronosyl transferases
VOO: virgin olive oil
WHO: World Health Organization
